# Cerebral oxygen monitoring in intensive care

**DOI:** 10.1186/cc9733

**Published:** 2011-03-11

**Authors:** G Hadjipavlou, O Touma

**Affiliations:** 1 Nuffield Department of Anaesthetics, Oxford, UK; 2 Oxford Radcliffe Hospitals, Oxford, UK

## Introduction

The purpose of this literature review is to look at the potential of cerebral oxygen monitoring in the intensive care setting and how this monitoring modality will impact our current practice.

## Methods

A PubMed literature search was conducted using the search items 'cerebral, oxygenation, and monitoring'. The search was limited to adults and the search items limited to the title or abstract. Articles selected were those that demonstrated a positive or negative benefit of cerebral oxygen monitoring on neurological outcome after surgery or intensive care.

## Results

The search revealed a total of 449 possible articles when conducted in December 2010. This was narrowed down to 18 articles related to monitoring cerebral oxygen. Patient outcomes: cerebral oxygen monitoring and the aggressive treatment of cerebral hypoxia reduced mortality and improved long-term outcomes after traumatic brain injury and coronary artery bypass surgery. Near-infrared spectroscopic cerebral oxygen monitoring is capable of detecting ischaemic cerebral perfusion deficits and may be more sensitive than transcranial Doppler in assessing blood flow and detecting delayed ischaemic deficits in subarachnoid haemorrhage. Cerebral hypoxia can persist despite good cerebral perfusion and normal intracranial pressure. Cerebral oxygenation monitoring can prevent iatrogenically driven hyperoxia and hyperperfusion, and can detect cerebral hypoxia before drops in standard pulse oximetry monitoring.

## Conclusions

The authors believe evidence is gathering suggesting that cerebral oxygen monitoring may play an important role in neurointensive and adult intensive care centres. Cerebral hypoxia worsens long-term neurological outcomes, and this modality has potential to help reduce morbidity.
